# Vitamin D in the Prevention and Treatment of Inflammatory Skin Diseases

**DOI:** 10.3390/ijms26115005

**Published:** 2025-05-22

**Authors:** Zrinka Bukvić Mokos, Lucija Tomić Krsnik, Kristijan Harak, Danijela Marojević Tomić, Deša Tešanović Perković, Marija Vukojević

**Affiliations:** 1 Department of Dermatology and Venerology, University Hospital Centre Zagreb, Kišpatićeva 12, 10000 Zagreb, Croatia; zrinka.bukvic.mokos@kbc-zagreb.hr (Z.B.M.); kristijan.harak@gmail.com (K.H.); mvukojevic97@gmail.com (M.V.); 2 School of Medicine, University of Zagreb, Šalata 3, 10000 Zagreb, Croatia; 3 Poliklinika Vaš Pregled, Trpinjska ulica 5A, 10000 Zagreb, Croatia; danijelamarojevic5@gmail.com; 4 Specijalna bolnica Agram, Trnjanska cesta 108, 10000 Zagreb, Croatia; tesanovicdesa@gmail.com

**Keywords:** vitamin D, psoriasis, atopic dermatitis, acne, hidradenitis suppurativa

## Abstract

Vitamin D, a hormone synthesized in the skin through ultraviolet B radiation (UVB), plays a crucial role not only in calcium and phosphate homeostasis but also in regulating skin homeostasis and modulating immune responses. In keratinocytes, vitamin D is converted to its active form, 1,25-dihydroxyvitamin D_3_ (1,25(OH)_2_D), which interacts with the vitamin D receptor (VDR) to regulate gene expression involved in proliferation, differentiation, and antimicrobial defense. Dysregulation of this pathway has been implicated in inflammatory skin diseases such as psoriasis, atopic dermatitis, acne vulgaris, and hidradenitis suppurativa. These conditions are associated with altered epidermal differentiation, immune imbalance, and microbial interactions, where vitamin D plays a modulatory role by suppressing proinflammatory cytokines, enhancing antimicrobial peptide synthesis, and restoring skin barrier integrity. Topical vitamin D analogues have shown therapeutic benefits in psoriasis, while emerging evidence supports the adjunctive role of vitamin D supplementation in acne, hidradenitis suppurativa, and atopic dermatitis. Despite promising associations between low serum vitamin D levels and disease severity, a causal relationship remains uncertain. This review integrates molecular mechanisms with clinical findings, emphasizing the role of vitamin D in cutaneous physiology and pathology, and highlights the need for further research into targeted supplementation strategies in dermatological disorders.

## 1. Introduction

Vitamin D is one of the oldest hormones on Earth and plays an essential pleiotropic role in maintaining global homeostasis [1]. Although the physiological role of vitamin D is most commonly associated with the maintenance of the musculoskeletal system, the biological properties of this relatively simple compound extend far beyond the regulation of calcium and phosphorus homeostasis [2]. In the past few decades, the extraskeletal effects of vitamin D have become more evident, including its roles in regulating cell proliferation, differentiation, and apoptosis and in immune modulation [3]. The main source of vitamin D is skin exposure to sunlight. Human skin serves both as the site of vitamin D synthesis and as a target organ for its biologically active form [4].

### 1.1. Vitamin D Synthesis and Functions

**Figure 1:**
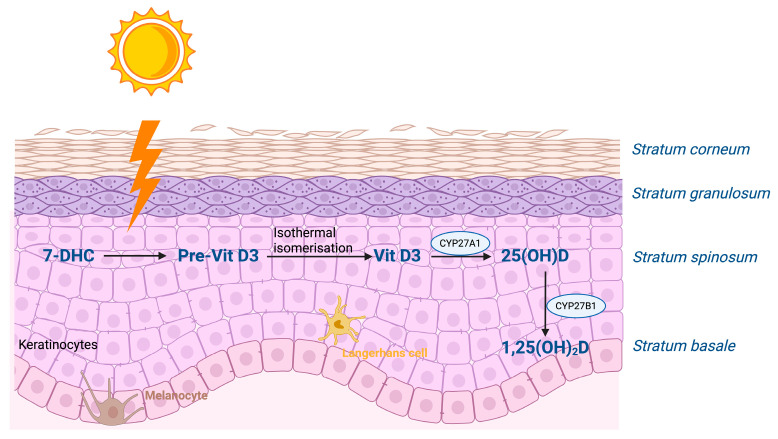
Vitamin D_3_ pathway in the human skin. When exposed to UVB radiation, vitamin D_3_ is produced in the skin from its precursor 7-DHC. It is converted to its active form, 1,25(OH)_2_D, through two hydroxylation steps carried out by enzymes CYP27A1 and CYP27B1. Abbreviations: UVB, ultraviolet radiation B; 7-DHC, 7-dehydrocholesterol; 25(OH)D_,_ 25-hydroxyvitamin D; 1,25(OH)_2_D_,_ 1,25-dihydroxyvitamin D; CYP27A1, 25-hydroxylase; CYP27B1, 1α-hydroxylase. The image was created with BioRender.com, https://biorender.com.

The epidermis is one of the most important sources of vitamin D for the body (Figure 1). Upon exposure to sunlight, i.e., ultraviolet (UV) radiation (action spectrum 290–315/280–320 nm or UVB), in the photochemical reaction, 7-dehydrocholesterol (7-DHC) is converted into pre-vitamin D_3_ in keratinocytes of the basal and spinous cell layers of the epidermis [3,4,5]. Pre-vitamin D_3_ is then converted into vitamin D_3_ (cholecalciferol) through thermal isomerization [6]. Following cutaneous synthesis, vitamin D_3_ enters the circulation primarily bound to vitamin D binding protein (VDBP). In contrast, after intestinal absorption, vitamin D_3_ is transported in association with both VDBP and lipoproteins [7]. Whether synthesized in the skin or acquired through the diet, vitamin D_3_ is biologically inactive and undergoes two subsequent hydroxylation reactions to gain its full hormonal activity. First, in the liver, the enzyme vitamin D 25-hydroxylase (CYP2R1) converts it into 25-hydroxyvitamin D (25(OH)D), also known as calcidiol. Then, in the kidney, the enzyme 1α-hydroxylase (CYP27B1) converts it into the active metabolite 1,25-dihydroxyvitamin D (1,25(OH)_2_D), also referred to as calcitriol. Both 25(OH)D and 1,25(OH)_2_D can be metabolically deactivated through hydroxylation by the enzyme 24-hydroxylase (CYP24A1) [4]. Vitamin D nutritional status is evaluated by measuring the serum 25(OH)D level, which is the predominant vitamin D metabolite in the bloodstream [5,8].

Aside from producing Vitamin D, keratinocytes, key cells in the epidermis, contain the enzymes CYP27A1 (25-hydroxylase) and CYP27B1 (1-hydroxylase), which are responsible for converting vitamin D_3_ into its active form, 1,25(OH)_2_D. Keratinocytes are the only cells in the body that can synthesize vitamin D_3_ from its precursor, 7-DHC, and convert vitamin D_3_ to its active metabolite, 1,25(OH)_2_D [9]. In parallel, several extracutaneous tissues, including the breast, colon, brain, ovaries, and prostate, also express 25-hydroxylase, enabling local conversion of vitamin D_3_ into 25(OH)D and supporting autocrine production of this metabolite within those tissues [7]. Keratinocytes, like many other cells, express the vitamin D receptor (VDR), allowing them to respond to locally produced 1,25(OH)_2_D in both an autocrine and paracrine manner [9]. 1,25(OH)_2_D is a high-affinity ligand of the VDR, which regulates the expression of hundreds of target genes by binding to the vitamin D response elements on the chromosome [10,11]. VDR is most active in keratinocytes during cellular differentiation and proliferation, regulating epidermal proliferation in the basal layer and promoting the gradual differentiation of keratinocytes as they form the upper layers of the epidermis [4,12].

Although initially not considered physiologically significant, the parent compound vitamin D_3_ plays a crucial role not only in classical endocrine functions but also in the autocrine, paracrine, and intracrine mechanisms of vitamin D actions [7]. While it is well established that vitamin D_3_ must be metabolized to 1,25(OH)_2_D to exert its biological effects, the conventional metabolic model has not fully recognized the importance of cellular accessibility to both the parent compound vitamin D_3_ and its primary circulating metabolite 25(OH)D [7]. Compared to 25(OH)D, vitamin D_3_ is more effectively internalized by most cell types, except those expressing the megalin–cubilin system, such as cells in the kidney and parathyroid gland. Most other cell types depend on their autocrine or paracrine environments; however, the availability of circulating 25(OH)D is limited due to its strong binding affinity for VDBP. In contrast, the parent compound vitamin D_3_ has a 10-to-12-fold lower binding affinity for VDBP, allowing a greater proportion of unbound (“free”) vitamin D_3_ to enter cells and undergo intracellular activation. This difference enhances the bioavailability of vitamin D_3_ for local conversion to its active form, particularly in tissues with limited access to tightly VDBP-bound 25(OH)D. Enhanced cellular access to vitamin D_3_ is functionally significant. Following oral administration of vitamin D_3,_ keratinocytes have been shown to upregulate antimicrobial peptides, such as cathelicidin, even after modest rises in circulating 25(OH)D concentrations, suggesting that vitamin D_3_ itself reaches the skin and is metabolized in situ [7].

### 1.2. Vitamin D and Epidermal Proliferation and Differentiation

**Figure 2:**
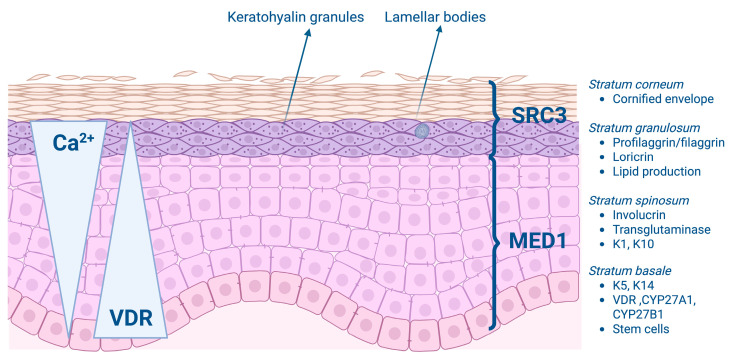
Epidermal layers and the role of VDR and its coactivators. The epidermis comprises four distinct layers, each with specialized functions regulated in part by the VDR and its coactivators. The basal layer of the epidermis, the stratum basale, contains stem cells, which, through epidermal proliferation, provide the cells for the upper layers. This layer also contains high levels of VDR and the enzymes CYP27A1 and CYP27B1, as well as keratins K5 and K14. Here, the VDR works closely with the coactivator MED1 to regulate cell proliferation. As keratinocytes migrate into the stratum spinosum, they begin to differentiate. In this layer, keratins K1 and K10 become prominent, and proteins like involucrin and transglutaminase are produced, which are essential for forming the cornified envelope. Next, in the stratum granulosum, terminal differentiation reaches its peak activity. Here, keratinocytes produce filaggrin and loricrin, key components of the cornified envelope. Lamellar bodies also form in this layer, secreting lipids into the space between the stratum granulosum and the stratum corneum to create a permeability barrier. The coactivator SRC3 becomes most active at this stage, partnering with VDR to regulate lipid processing, lamellar body formation, and the expression of antimicrobial peptides like cathelicidin and CD14—critical for innate immunity. Finally, the stratum corneum forms the outermost layer, composed of fully differentiated, enucleated keratinocytes embedded in a lipid matrix. This layer provides both a physical barrier and immune protection. From basal stem cell to fully differentiated corneocyte, 1,25(OH)_2_D-VDR signaling guides keratinocyte behavior, shifting from MED1 coactivators in the early stages to SRC3 in later stages. This dynamic regulation ensures proper epidermal development, barrier formation, and immune defense. Abbreviations: vitamin D receptor, VDR; CYP27A1, 25-hydroxylase; CYP27B1, 1α-hydroxylase; MED1, Mediator complex 1; SRC 3, Steroid Receptor Coactivator 3; 1,25(OH)_2_D, 1,25-dihydroxyvitamin D. The image was created with BioRender.com, https://biorender.com.

The epidermis consists of four layers of keratinocytes, each at a different stage of differentiation [4]. Epidermal differentiation is a complex, sequential, tightly regulated process (Figure 2) [13]. The basal layer, or stratum basale, rests on the basal lamina (the layer that separates the dermis from the epidermis) and consists of cylindrical keratinocytes along with stem cells whose primary function is the production of transient amplifying cells, which begin the differentiation process as they ascend into the spinous layer [4,12]. Keratinocytes in the basal layer primarily express the keratin pair keratin 5 and 14 [4]. The stratum spinosum, the layer above the stratum basale, contains cells that initiate the production of the keratins K1 and K10, which are characteristic of more differentiated layers of the epidermis. Moreover, precursor molecules of the cornified envelope, such as involucrin, appear in the stratum spinosum, along with the transglutaminase K, an enzyme responsible for the ε-(γ-glutamyl) lysine cross-linking of these substrates into the insoluble cornified envelope [4]. Above the stratum spinosum is the granular layer, or stratum granulosum, which is characterized by electron-dense keratohyalin granules that contain profilaggrin, a precursor of filaggrin, and loricrin, which is an essential component of the cornified envelope. Filaggrin facilitates keratin filament aggregation and, when metabolized into smaller fragments, is also believed to contribute to the hydration of the permeability barrier. Filaggrin mutations are often found in patients with atopic dermatitis [4]. Lamellar bodies, containing both long-chain fatty acids and antimicrobial peptides from the stratum granulosum, line the membrane that separates the stratum granulosum from the stratum corneum [14]. Lamellar bodies are positioned to release their contents into the extracellular space, where the lipids and antimicrobial peptides play a role in maintaining the permeability barrier and defending against microorganisms [15].

As keratinocytes transition from the stratum granulosum to the stratum corneum, they undergo destruction of their organelles with further maturation of the stratum corneum into an insoluble, highly resistant structure that surrounds the keratin–filaggrin complex and is associated with an extracellular lipid milieu [4].

1,25(OH)_2_D, through its receptor VDR, regulates every step of the differentiation process. In vivo and in vitro studies have demonstrated that vitamin D affects keratinocyte proliferation and differentiation in a dose-dependent manner. Interestingly, low concentrations (≤10^−9^ m) of vitamin D promote keratinocyte proliferation in vitro, whereas high concentrations (>10^−8^ m) inhibit proliferation and promote differentiation [16,17]. 1,25(OH)_2_D promotes the production of keratin 1, involucrin, and transglutaminase in the stratum spinosum, which helps maintain proper barrier function [12]. Furthermore, it induces the synthesis of filaggrin, loricrin, antimicrobial peptides, and long-chain fatty acids and the formation of the cornified envelope in the stratum granulosum [4,12,18]. The process of epidermal differentiation and its regulation by 1,25(OH)_2_D and VDR is sequential, with different genes and pathways being activated in keratinocytes at various stages of differentiation. VDR and CYP27B1 are expressed throughout the epidermis, with the highest level of expression in the stratum basale [19]. The transcriptional activity of VDR is tightly regulated by a number of co-regulators that can show cell specificity [11,20]. The major co-activator complexes in the epidermis regulating VDR function are the Mediator complex (previously known as DRIP or VDR interacting proteins), of which MED1 is the major VDR-binding component, and the Steroid Receptor Coactivator (SRC) complex, of which SRC2 and SRC3 are the major VDR-binding components [21]. MED1 and SRC3 are expressed in a reciprocal pattern in the epidermis. MED1 is primarily present in the stratum basale, while SRC3 is localized in more differentiated layers, specifically the stratum granulosum [21]. In undifferentiated keratinocytes, MED directly interacts with VDR to inhibit its activity, whereas in the differentiated cell layers of the stratum granulosum, co-activators SRC2 and SRC3 regulate VDR activity, enhancing its function [22]. The knockdown of MED1 in the epithelium leads to increased keratinocyte proliferation and disrupts the expression of keratins 1 and 10 and involucrin [18]. In contrast, SRC3 knockdown reduces glucosylceramide production and impairs lamellar body formation [22]. Both co-activators enhance vitamin D-induced transcription in proliferating cells and are differentially involved in vitamin D-driven differentiation of epidermal keratinocytes. SRC3 regulates terminal differentiation, while MED1 is involved in the regulation of proliferation and early keratinocyte differentiation [21].

In addition to its role in epidermal differentiation, 1,25(OH)_2_D enhances the innate immune function of keratinocytes by stimulating the expression of toll-like receptor (TLR2) and its co-receptor CD14. This activation triggers a feedback loop in which TLR2 and CD14 induce CYP27B1 expression, leading to the increased production of 1,25(OH)_2_D. In turn, this promotes the synthesis of cathelicidin, a potent antimicrobial peptide [23,24].

It is important to highlight the role of calcium gradient in the keratinization process. In vivo, there is a gradient of increasing intracellular Ca^2+^ concentration across the epidermal layers, with the peak concentration occurring in the stratum granulosum [25]. Calcium concentration below 0.07 mM promotes keratinocyte proliferation, whereas acutely raising extracellular calcium levels above 0.1mM (calcium switch) induces the rapid redistribution of several proteins from the cytosol to the membrane, where they participate in epidermal differentiation. These proteins include the calcium receptor (CaR), phospholipase C-γ1(PLC-γ1), Src kinases, various catenins such as β-catenin, phosphatidyl inositol 4-phosphate 5-kinase 1α (PIP5K1α), and the formation of the E-cadherin/catenin complex (adherens junctions) with phosphatidyl inositol 3 kinase (PI3K). These proteins play important roles in calcium and vitamin D-induced epidermal differentiation [26,27,28]. Within hours of the calcium switch, keratinocytes begin producing keratins K1 and K10, followed by increased levels of profilaggrin, involucrin, and loricrin. These proteins, along with others, are cross-linked into insoluble cornified envelope by the calcium-induced transglutaminase, marking the final step in the differentiation process [29].

The CaR is crucial for these calcium-induced responses, and its expression is upregulated by 1,25(OH)_2_D, which increases the keratinocytes’ sensitivity to calcium prodifferentiation effects [30]. Deletion of CaR in keratinocytes disrupts their response to extracellular calcium by reducing calcium stores and inhibiting the formation of the E-cadherin/catenin complex [26,27]. Mice without the CaR exhibit a defective permeability barrier caused by the abnormal production of essential lipids and proteins necessary for barrier formation, along with an impaired innate immune response. Furthermore, CaR deletion leads to the reduced expression of VDR and CYP27B1, likely contributing to the impaired epidermal differentiation observed in CaR-deficient mice [31]. As mentioned earlier, 1,25(OH)_2_D induces CaR, and just as 1,25(OH)_2_D/VDR stimulates CaR expression, calcium/CaR is essential for VDR and CYP27B1 expression. This highlights the strong interaction between calcium and vitamin D signaling in epidermal differentiation. Consequently, 1,25(OH)_2_D/VDR enhances the keratinocyte response to calcium’s prodifferentiation effects, while CaR’s role in the regulation of VDR and CYP27B1 expression further supports the prodifferentiation actions of 1,25(OH)_2_D [31]. The synergism between CaR and VDR is clearly demonstrated by their joint regulation of the expression of several genes, including a member of the PLC family [32]. All members of the PLC family are induced both by 1,25(OH)_2_D and calcium, and inhibition of PLC-γ1 expression blocks the differentiation induced by 1,25(OH)_2_D and calcium [32]. Another significant synergistic effect of calcium and 1,25(OH)_2_D in epidermal differentiation is their ability to induce involucrin and transglutaminase, which is due to the close proximity of the calcium response element and vitamin D response element in the involucrin promoter [33].

Understanding the regulatory pathways and molecular mechanisms through which vitamin D influences the skin is essential. Human skin is inseparably connected to vitamin D, serving both as the site of synthesis and a primary target for its action. In this review, we provide a comprehensive overview of current knowledge regarding the role of vitamin D in skin inflammatory diseases, including atopic dermatitis, acne vulgaris, psoriasis vulgaris and hidradenitis suppurativa.

## 2. Vitamin D and Psoriasis

Psoriasis is a chronic, immune-mediated inflammatory disorder affecting about 2–3% of the global population [34]. It is caused by immune dysfunction that leads to continuing inflammation, increased proliferation of skin cells, and the formation of erythemato-squamous plaques [34]. Around 80% to 90% of cases are plaque-type psoriasis, but there are also less common forms like guttate, pustular, inverse, and erythrodermic. Psoriatic patches typically appear on the scalp, elbows, knees, hands, feet, nails, and genitals. They are commonly associated with itching and thickened or pitted nails [35]. An overactive immune system plays a central role in the development of skin lesions [35,36]. Although the exact cause is not completely clear, psoriasis is thought to result from a combination of genetic predisposition and environmental triggers like infections, skin injuries, stress, smoking, or UV exposure [36]. In people with a genetic tendency toward psoriasis, these factors can activate the immune system, leading to inflammation and excessive skin cell growth [37]. On a cellular level, psoriasis is linked to activated T cells and a disrupted balance between Th1 and Th2 immune responses [38]. In healthy individuals, Th1 cells release pro-inflammatory cytokines like IL-2, IFN-γ, and IL-12, while Th2 cells are more involved in anti-inflammatory responses, producing IL-4 and IL-10. In patients with psoriasis, the immune balance shifts toward a Th1-dominant state, leading to an overproduction of cytokines such as IL-2 and IFN-γ, along with a rise in Th17 and Th22 cell activity [36,38]. Th17 cells produce cytokines such as IL-6, IL-17, and IL-22, which help drive inflammation and rapid skin cell growth seen in psoriasis, leading to the development of plaques [36,38]. Moreover, innate immune cells like dendritic cells, macrophages, neutrophils, natural killer cells, and even keratinocytes also play a significant role in psoriasis by producing proinflammatory cytokines, which help intensify the immune response [36]. When dendritic cells are activated by self-DNA and peptides such as LL-37, they produce interferons, which cause the inflammatory response and enhance Th1 and Th17 activity. The inflammation causes keratinocyte hyperproliferation and leads to immune cell migration and neoangiogenesis, forming new blood vessels in affected skin areas [36]. Pro-inflammatory cytokines like TNF-α, IL-17A/F, and IL-22 play a major role in psoriasis. Therefore, treatments that target these molecules have transformed therapy for moderate-to-severe cases. Biologic drugs that block IL-17, IL-23, or TNF-α suppress inflammation by interrupting the signals that promote keratinocyte proliferation and immune system overactivation [36].

### The Association Between Vitamin D and Psoriasis

Vitamin D, particularly in its active form 1,25(OH)_2_D, plays a significant role in immune modulation and skin homeostasis [4]. Vitamin D is known to act on both the innate and adaptive immune systems, so it plays a valuable role in treating psoriasis. Active vitamin D promotes the function of immune cells such as dendritic cells, T-cells, and macrophages by enhancing their phagocytic property and stimulating the production of antimicrobial peptides, such as cathelicidin, which contribute to the body’s defense against infections [39]. Within the adaptive immune system, vitamin D helps reduce inflammation by lowering the activity of Th1 cells by blocking the production of IL-2 and IFN-γ and promoting Th2 cell differentiation. This shift supports the release of anti-inflammatory cytokines like IL-4 and IL-10, which help reduce the immune response [40]. Vitamin D regulates keratinocyte differentiation and proliferation by activating VDR, which supports the production of important barrier proteins like loricrin and filaggrin [36,41]. This supports skin integrity and helps slow the rapid cell turnover that occurs in psoriatic plaques. Hosomi et al. were the first to show that 1,25(OH)_2_D encourages keratinocyte differentiation by inhibiting DNA synthesis [42]. This led to a reduced number of cells, increased cell size and density, differentiation into squamous, enucleated cells, and enhanced cornified envelope formation.

Topical vitamin D and its analogues are widely used as first-line treatments for mild to moderate psoriasis due to their effectiveness and safety profile [43]. Calcipotriol, introduced in the late 1980s, continues to be one of the most widely used treatments for psoriasis. Interestingly, it has been shown to reduce IL-6 expression in psoriatic lesions, yet it appears to have little or no effect on TNF-α levels, suggesting a more targeted mechanism of action. When used alongside treatments such as UVB phototherapy, fumaric acid esters, cyclosporine, or acitretin, calcipotriol has been found to improve overall therapeutic outcomes [44,45,46,47]. Clinical trials have demonstrated that the fixed-dose combination of calcipotriene (0.005%) and betamethasone dipropionate (0.064%) is highly effective, offering improved skin clearance with few adverse effects [48]. Tacalcitol is another vitamin D analogue that is both effective and well tolerated in long-term treatment. Extended application of ointments containing 4–20 µg/g of tacalcitol has shown significant improvements in PASI scores, as well as good patient tolerance and stable hormonal and calcium levels [49]. Tacalcitol enhances the effect of NB-UVB phototherapy, highlighting its usefulness as a supportive treatment [50]. Maxacalcitol, a more potent vitamin D3 analogue, has demonstrated greater efficacy than calcipotriol and tacalcitol in laboratory settings, promoting keratinocyte differentiation and limiting cell proliferation, avoiding hypercalcemia [51,52]. Trials have verified its therapeutic importance across different dosages, with even better outcomes when paired with NB-UVB [53]. However, the potential for hypercalcemia is higher compared to other analogues, which may limit its use in routine care [54]. The regulatory role of 1α,25-dihydroxyvitamin D_3_ in cell proliferation and keratinocyte differentiation was observed in three separate in vitro studies [42,55,56].

Several studies have demonstrated a consistent association between low serum 25-hydroxyvitamin D (25(OH)D) levels and psoriasis [57,58,59,60,61,62,63]. For example, Chandrashekar et al. and Maleki et al. found that individuals with psoriasis had notably reduced concentrations of 25-hydroxyvitamin D compared to healthy controls. These findings also revealed a negative association with PASI scores, suggesting that lower vitamin D levels are linked to more severe disease manifestations [57,60]. Bergler-Czop et al. and Pokharel et al. had similar findings in patients with psoriasis [58,61]. These data were further supported by meta-analyses, which consistently show lower 25(OH)D levels in individuals with psoriasis and a strong inverse link to PASI scores [62]. However, not all studies agree; some have found no meaningful differences in vitamin D levels between psoriatic patients and healthy controls [60,63,64]. Upon examining the available research, it remains unclear whether vitamin D deficiency is a contributing factor to the development of psoriasis or a consequence of the disease [65].

The first oral form of vitamin D used in psoriasis treatment was 1α(OH)D, noted for its ability to reduce keratinocyte overgrowth and modify keratin expression [66]. Trials involving higher daily doses (ranging from 5000 to 50,000 IU) have shown encouraging results, with improvements in PASI scores and reductions in inflammatory markers without evident toxicity [67]. Still, the outcomes have not been universally positive. For instance, studies by Ingram et al. and Jarrett et al. using monthly doses of 100,000 IU demonstrated elevated vitamin D levels but failed to produce meaningful clinical improvements [63,64]. Similarly, research by Prystowsky et al. found no additional benefit when oral calcitriol was combined with UVB phototherapy [67]. Gumowski-Sunek et al. observed alterations in calcium metabolism linked to oral calcitriol, a side effect not seen with equivalent topical doses of calcipotriol [68].

The variability in findings across studies may be affected by differences in dose, duration, and formulation of vitamin D analogues. Heterogeneity among followed patients in terms of disease severity, baseline vitamin D status, genetic background, immune function, and presence of comorbidities may also impact the results. For instance, Pokharel et al. identified an inverse relationship between serum vitamin D levels and disease severity, but those correlations did not translate into therapeutic efficacy [61].

Vitamin D is supposed to effect psoriasis through its regulation of keratinocyte proliferation and differentiation, as well as T-cell response. However, in chronic or severe cases, the modulation of T-cell response might not beneficial enough, due to established immune dysregulation and evolved keratinocyte proliferation. Since clinical trials to date are taking in account only individuals with established moderate or severe psoriasis, it leaves a question of potential preventive or early-stage therapeutic effects of vitamin D. This highlights a significant gap in the literature regarding the timing and context in which vitamin D supplementation might offer the greatest benefit.

In summary, although high-dose oral supplementation may offer therapeutic value, topical vitamin D formulations continue to be the safer and more widely favored option. New analogues that specifically target the skin’s vitamin D pathways represent a promising direction for future psoriasis treatments.

Recent studies on the association between vitamin D serum levels and psoriasis are presented in Table 1.

## 3. Vitamin D and Atopic Dermatitis

Atopic dermatitis (AD), also termed atopic eczema, represents a prevalent, chronic, and recurrent inflammatory dermatosis [70]. It is a complex disease with a spectrum of clinical presentations and combinations of symptoms. AD typically presents in infancy or early childhood and usually shows partial or complete remission over time. However, the disease persists into adulthood in approximately 10% of patients, showing a more severe phenotype associated with a higher disease burden. These patients frequently experience not only pronounced cutaneous symptoms but also significant impairment in quality of life. The clinical manifestations of AD are heterogeneous and may include xerosis, erythema, excoriations, edema, serous exudate, erosions, crusting, and lichenification, with considerable interindividual variability in presentation and severity [71]. AD affects up to 20% of the pediatric population and up to 3% of adults; recent evidence shows a continuing rise in its prevalence, particularly in low-income countries [72].

The pathophysiology of AD is complex and multifactorial. Still, it can generally be divided into three major categories, each modulated by genetic and environmental factors: epidermal barrier dysfunction, immune dysregulation, and microbiome alteration [73]. Filaggrin, a major epidermal protein, is essential for the structure and function of the stratum corneum, the outermost layer of the skin, and is recognized as a key factor in the pathogenesis of AD. The stratum corneum provides a physical barrier, serving as the first line of defense against environmental factors, pathogens, and allergens, while also maintaining water homeostasis [74]. Loss-of-function mutations in filaggrin have been implicated in severe AD due to a potential increase in trans-epidermal water loss (TEWL), pH alterations, and dehydration [75].

Furthermore, there is an imbalance in immunity involving Th1, Th2, and Treg cells, culminating in alterations in Th1- and Th2-mediated immune responses and IgE-mediated hypersensitivity. The expression levels of the Th2 cytokines IL-4 and IL-13 are elevated in AD lesions [76]. These cytokines reduce epidermal differentiation, contributing to reduced filaggrin expression and antimicrobial peptide expression. IL-31 induces severe pruritus in addition to its inhibitory effects on epidermal differentiation. The immune system in AD becomes more heterogeneous and complex with the activation of other immune cells, such as Th22 and Th17 cells [77].

Vitamin D has an important role in maintaining normal skin physiology and regulating immune function. It contributes to the formation of the cornified envelope and supports the synthesis of the lipid permeability barrier essential for epidermal integrity. Additionally, vitamin D stimulates the expression of human cathelicidin, an antimicrobial peptide known to be deficient in AD patients [78,79,80]. Given that the pathogenesis of AD involves both impaired epidermal barrier function and immune dysregulation—processes in which vitamin D plays a regulatory role—it is biologically plausible that vitamin D status may influence the risk for AD development and its severity [80].

### 3.1. The Role of Vitamin D in the Pathophysiology of Atopic Dermatitis

Vitamin D exerts its effects primarily through the VDR, which is expressed in various immune cells, including keratinocytes, T cells, and monocytes. The interaction between vitamin D and the VDR modulates the transcription of numerous genes implicated in immune regulation and cutaneous homeostasis. The biologically active form of vitamin D, calcitriol (1,25-dihydroxyvitamin D), facilitates keratinocyte differentiation and upregulates the synthesis of key antimicrobial peptides (AMPs), including cathelicidin and defensins, which are essential components of the skin’s innate immune defense [81]. These peptides are crucial in protecting the skin from infections, a frequent complication in patients with AD.

Vitamin D is involved in important regulatory mechanisms of the innate and adaptive immune system. VDR has been found in various cells, including keratinocytes and numerous immune system cells. 1,25-dihydroxyvitamin D (1,25(OH)_2_D) exerts immunomodulatory effects by suppressing the proliferation of T helper 1 (Th1) cells, which may produce interferon-gamma, IL-2, and activating macrophages. It also inhibits T helper 17 (Th17) cells, which are capable of secreting interleukins IL-17 and IL-22 [82,83,84,85]. Moreover, 1,25(OH)_2_D promotes the expansion of regulatory T cells (CD4^+^/CD25^+^), enhancing the production of the anti-inflammatory cytokine IL-10, further downregulating Th1 and Th17 responses. Additionally, it has been shown that serum vitamin D levels and components involved in its metabolism, such as VDR and GC gene SNPs, may be related to regulating the immune response and the expression of atopic diseases [86,87].

### 3.2. The Association Between Serum Levels of Vitamin D and Atopic Dermatitis

A number of biologically plausible mechanisms support an inverse relationship between serum vitamin D concentrations and the manifestation of atopic dermatitis (AD), particularly concerning disease severity. Human keratinocytes possess the enzymatic apparatus to produce calcitriol, the active compound of vitamin D, from the precursor 7-dehydrocholesterol under the influence of ultraviolet (UV) B radiation [88,89]. In in vitro studies, vitamin D_3_ (calcitriol) has been shown to induce cathelicidin expression in keratinocytes, which enhances antimicrobial activity against *S. aureus* and selectively reduces cutaneous lymphocyte-associated antigen expression. It has been demonstrated that cathelicidin does not influence lymphocyte migration patterns to other tissues, thus specifically decreasing T-lymphocyte homing into the skin [24,90,91]. The clinical benefits observed were biologically plausible due to the known actions of vitamin D on skin physiology and immune function.

Evidence from epidemiological, clinical, and immunological studies suggests an association between serum vitamin D levels and AD development. Lower circulating levels of vitamin D have been consistently observed to inversely correlate with the incidence and severity of AD [77,92,93,94,95,96]. Recent research has reported that low vitamin D status at birth is associated with a higher risk of AD during infancy [92]. Furthermore, elevated maternal vitamin D intake during pregnancy has been associated with a reduced risk of developing wheezing disorders and atopic eczema, suggesting a potential protective role in early immune and skin barrier development [97]. Moreover, observational studies have revealed a correlation between low serum vitamin D levels and increased AD severity [90,98]. In addition, systematic review and meta-analysis found that vitamin D deficiency is commonly associated with exacerbations in AD, indicating that patients with lower 25-hydroxyvitamin D levels presented with more severe forms of the disease [99,100]. Research involving children particularly showcases the benefits of vitamin D in alleviating atopic dermatitis symptoms. For instance, Munawwarah et al. found that higher serum levels of vitamin D correlated inversely with AD severity, suggesting that vitamin D could be protective in developing the disease [101]. These findings highlight the importance of assessing serum vitamin D levels in AD patients, particularly those who did not benefit from standard treatment [102]. Nevertheless, this association has not been confirmed by further studies, suggesting that the relationship may be more complex than previously understood [80]. The results of selected clinical studies evaluating the association of serum vitamin D levels with the occurrence and severity of AD are shown in Table 2.

On the contrary, several studies reported elevated serum vitamin D levels in patients with AD, putting into debate whether vitamin D deficiency represents a consequence rather than a risk factor in AD pathogenesis [88]. Despite this ongoing debate, maintaining sufficient vitamin D status is still considered potentially beneficial as part of a comprehensive therapeutic strategy [105,106].

The clinical efficacy of vitamin D supplementation in managing atopic dermatitis has been evaluated in various studies, revealing promising outcomes. For instance, a randomized controlled trial conducted by Mansour et al. demonstrated that vitamin D supplementation significantly improved clinical outcomes, including reduced severity scores as measured by the eczema area and severity index (EASI) in patients with severe AD [107]. Similarly, a systematic review and meta-analysis by Kim et al. reported consistent findings where vitamin D supplementation led to a statistically significant decrease in the Severity Scoring of Atopic Dermatitis (SCORAD) outcomes across several trials [108]. Selected clinical studies evaluating the effects of vitamin D supplementation on the severity of AD are shown in Table 3.

Consequently, it was suggested that topical treatment or oral supplementation of vitamin D might improve barrier function and, thus, inflammation in AD. One of the few studies investigating this hypothesis showed increased cathelicidin expression in skin biopsies in patients suffering from AD after oral vitamin D supplementation [112].

Findings of a large number of studies investigating the association between vitamin D and atopic dermatitis are often contradictory. The leading cause of this variability lies in significant methodological differences across studies, including variations in study design, sample size, follow-up duration, and inclusion and exclusion criteria for participants. Furthermore, atopic dermatitis is a multifactorial disease, with different genetic and environmental factors other than vitamin D playing significant roles in its pathogenesis.

A particular issue is the lack of standardization in supplementation protocols, such as the dosage and duration of vitamin D administration and the timing of supplementation, whether used preventively or therapeutically at various stages of the disease.

As mentioned, further research is necessary, with clearly defined methodological criteria, standardized supplementation protocols, and well-established clinical endpoints. Only through such an approach can we gain a more reliable understanding of the potential role of vitamin D in the prevention and treatment of atopic dermatitis.

## 4. Vitamin D and Acne

Acne is an inflammatory disorder of the pilosebaceous unit with a chronic and relapsing course. According to the Global Burden of Disease study, acne vulgaris is the eighth most prevalent disease globally, affecting as much as 9,38% of the global population [113]. It is estimated that 85% of people affected by acne are of adolescent age [114]. Acne eruptions are clinically manifested as non-inflammatory closed and open comedones or inflammatory lesions, such as papules, pustules, and nodules [115]. Regions of the body with the highest density of pilosebaceous units—such as the face, neck, chest, upper back, and arms—are the primary sites for the development of acne lesions. Available treatment options for acne vulgaris are manifold: topical agents, systemic antibiotics, oral isotretinoin, and hormonal agents, which can be used separately or in combination.

Four checkpoints of acne pathophysiology include excessive sebum production, follicular occlusion, *Cutinebacterium acnes* colonization, and release of inflammatory mediators. Interaction of those mechanisms results in the formation of microcomedone, the precursor of acne lesions. Apart from these central features of pathogenesis, external factors, called the exposome, also influence the occurrence of acne vulgaris. Dreno et al. classified exposomal factors into six main categories: nutrition, medication, occupational factors including cosmetics, pollutants, climatic factors, and psychological and lifestyle factors [116].

### 4.1. The Role of Vitamin D in Acne Pathogenesis

Vitamin D influences the main pathogenesis steps of acne vulgaris. Alterations in both sebum quantity and quality are involved in the pathogenesis of acne vulgaris, and these processes take place in sebocytes [117,118]. Sebocytes are stimulated by several pathways, with the central route being the IGF-1 signaling pathway [119]. When the sterol regulatory binding protein-1 (SREBP-1) is stimulated by IGF-1 and insulin, the PI3-K/Akt and the MAPK molecular pathway activate, subsequently leading to increased lipogenesis in sebocytes [120]. The abovementioned sebum modifications also result in *C. acnes* overgrowth, causing the activation of TLR-2 receptor expressed on sebocytes, inducing an inflammatory response mediated by IL-6, IL-8, IL-12, IL-1α, matrix metalloproteinase (MMP), and tumor necrosis factor-α (TNF-α) [119,121,122,123]. Sebocytes express an intranuclear VDR that binds a biologically active form of vitamin D, calcitriol. Krämer et al. proved that incubation of sebocytes with 1,25(OH)_2_D significantly reduced IL-6 and IL-8 secretion, therefore exhibiting potent anti-inflammatory effects of vitamin D on human sebocytes. Also, they demonstrated an inhibition of sebaceal cell growth in vitro at high pharmacologic concentrations of 1,25(OH)_2_D [124]. Likewise, Lee et al. demonstrated reduced production of IL-6, IL-8 and MMP-9 in sebocytes treated with 1,25-dihydroxyvitamin D_3_, and increased MMP-1 and MMP-3 in the treated cells [125]. Furthermore, 1,25(OH)_2_D exhibited potential for suppressing lipogenesis by reducing triglyceride accumulation in hamster sebocytes [126]. Thus, vitamin D supplementing could attenuate the inflammation generated in sebocytes, as well as sebaceous hyperplasia and lipogenesis, reducing acne eruptions.

Hyperkeratosis and hyperplasia of the follicular epithelium are the mainstays of comedogenesis [127]. Keratinocytes release IL-1α as an inflammatory response to the stimulation of the IL-1 receptor. This acts as one of the signals for the hypercornification of the infundibulum, and therefore, the overproduction of IL-1α takes part in acne pathogenesis [128,129,130]. Changes in sebum quality, such as an increase of unsaturated fatty acids in sebum, contribute to abnormal keratinization and lead to consequential follicular occlusion [131]. Vitamin D could take part in mediating hyperkeratosis and, therefore, reduce the formation of microcomedo. As sebocytes, keratinocytes also express a vitamin D receptor that binds calcitriol [132]. 1,25(OH)_2_D binds to the VDR on the keratinocytes and inhibits the transcriptional activity of β-catenin, thus inhibiting keratinocyte proliferation and promoting keratinocyte differentiation [133]. Hayashi et al. showed a potent comedolytic effect of topically applied maxacalcitol, an active vitamin D_3_ analogue, on pseudocomedones in rhino mice. It is suggested that the normalization of cornification is the basis of its comedolytic effects [134].

The interaction of *C. acnes* and TLR-2 and -4 receptors expressed on keratinocytes, sebocytes, and monocytes causes an inflammatory cascade through the NF-κB pathway, resulting in the release of proinflammatory cytokines including IL-6, IL8, IL-12, TNF- α, and MMP-9 [135,136,137,138]. When *C. acnes* overgrows in the pilosebaceous unit, it enters into conjunction with myeloid cells and sebocytes, triggering the NLRP-3 inflammasome, therefore inducing the release of the pro-inflammatory cytokine IL-1β [139,140,141]. It has been shown that topical vitamin D therapy significantly reduces the content of IL-1β in acne lesions and the number of inflammatory lesions [142]. Moreover, IL-1β and IL-6 are crucial for differentiating CD4+ cells to Th17 cells that secrete IL-17, further deepening the inflammatory response to *C. acnes* [143,144,145]. Agak et al. demonstrated that vitamin D and vitamin A caused the inhibition of IL-17 mRNA and its protein expression while not affecting IL-17 receptor gene expression [144]. Similarly, Ikeda et al. exhibited that 1,25-dihydroxy vitamin D_3_ and all-trans retinoic acid had synergistic suppressive effects on Th17 cells by reducing the mRNA expression of IL-17A and IL-22 [146]. Some studies showed a negative correlation between IL-17 and vitamin D levels in serum and lesions in acne patients, while being related to disease severity [147,148]. That being said, vitamin D could be effective as an adjuvant treatment for acne because of its immunomodulatory properties.

Nutrition is by far the most researched extrinsic factor in correlation with acne, and it is known that adequate nutrition and supplementation with vitamins, including vitamin D, could alleviate acne eruptions. Lim et al. compared levels of vitamin D in patients with and without acne, finding a higher prevalence of vitamin D deficiency in acne patients (48.8%) compared to healthy individuals (22.5%), with no notable differences in the mean serum concentration of vitamin D. In the same study, it was concluded that the 25-hydroxyvitamin D_3_ deficiency was inversely correlated with acne severity [149]. As mentioned earlier, Singh et al. also found a negative correlation between acne severity and vitamin D levels. In this study, there was no statistically significant difference in the mean 25-hydroxyvitamin D_3_ concentrations, but the prevalence of vitamin D deficiency in acne patients was higher (28%) in comparison to controls (6,7%) [148]. Likewise, Kemeriz et al. discovered a higher prevalence of vitamin D deficiency in the acne group (77.6%) in contrast to the control group (63.9%) and a statistically significant correlation between serum vitamin D levels and acne severity. They also found significant distinctions in serum vitamin D levels between groups [150]. Abd-Elmaged et al. detected similar findings, determining the lower serum levels of vitamin D in acne patients, as well as a significant negative correlation between the acne severity and serum 25-hydroxyvitamin D_3_ levels [147].

On the contrary, while finding significantly lower vitamin D levels in acne patients compared to controls, Alhetheli et al. found no correlation between vitamin D deficiency and the severity of the disease [151]. Toossi et al. found no significant difference in median serum vitamin D concentrations nor the prevalence of hypovitaminosis D in acne patients and controls. They also found no relation between disease severity and vitamin D levels [152]. Likewise, El-Ramly et al. showed no significant distinction between serum levels of 25-hydroxyvitamin D_3_ in acne patients and healthy controls and no correlation between acne severity and vitamin D status [153]. These findings are presented in Table 4.

### 4.2. The Effects of Vitamin D Supplementation on Acne

Few studies support the idea of vitamin D supplementation being beneficial to acne patients. The earlier mentioned Lim et al. evaluated the therapeutic potential of supplementation with vitamin D, finding a significant clinical improvement in acne patients who consumed 1000 IU of cholecalciferol per day [149]. Likewise, Ahmed Mohamed et al. gained positive results in acne patients with daily oral administration of vitamin D in a dose of 0.25 μg for 3 months. The researchers assessed the severity of acne using the GAGS scale and found significant clinical improvement in the severity, while the placebo group did not show compelling differences [155]. Ruikchuchit et al. conducted a randomized, double-blind, placebo-controlled study to evaluate vitamin D2 (ergocalciferol) therapy as an adjunction to topical benzoyl peroxide (BPO) administration. The participants received 40,000 IU weekly for 12 weeks in total while simultaneously applying topical BPO. The mean total lesion count decreased in both placebo and vitamin D groups, and there was no significant difference between the two groups. However, after the discontinuation of vitamin D and BPO, there was a continuous reduction of inflammatory lesions in the vitamin D group, while there was an increase of inflammatory lesions in the placebo group. These findings reinforced the concept of using vitamin D supplements as an additional anti-inflammatory treatment for acne [156].

The abovementioned studies supporting the hypothesis regarding the beneficial effect of vitamin D supplementation on reducing acne have several limitations. Small sample sizes and short study durations may reduce the accuracy of the conclusions. Furthermore, vitamin D is not the only factor related to the presence or severity of acne. Indeed, acne is a multifactorial skin disease, dependent on various intrinsic and extrinsic factors. Lack of control over other potential confounding factors, such as diet, hormones, or psychological stress, may have altered the final results of these studies. Overall, although vitamin D may have a potential supplementary role in acne treatment, its true benefits in this disease are yet to be clearly defined.

## 5. Vitamin D and Hidradenitis Suppurativa

Hidradenitis suppurativa (HS), also known as acne inversa, is a chronic, inflammatory skin disease affecting the hair follicles, characterized by the formation of painful, inflamed lesions, most commonly occurring in the axillary, inguinal, and anogenital regions. It most commonly develops in the early 20s. Diagnosis is made clinically, and the primary positive criterion is a history of recurrent painful or suppurating lesions in predilection sites occurring more than twice in six months [157,158]. Studies report the prevalence of HS ranging from 0.05% to 4% [159,160,161]. Given the studies’ different populations and research methods, the prevalence rate of 1% is generally accepted [162].

The clinical course is variable. Papules, pustules, and nodules appear in milder presentations of the disease, while in more severe cases, deep abscesses, sinus tracts, and scars usually affect multiple regions [162]. There are several scoring systems used to assess the severity of HS. The Hurley staging system is most commonly used in daily practice and recognizes three stages of the disease: I (single or multiple abscesses, no sinus tracts or scars), II (separated lesions, recurrent abscesses, sinus tracts, and scars), and III (extensive clinical presentation with numerous confluent sinus tracts and abscesses) [157]. It is useful for routine work and treatment choice, but it does not assess the extent of inflammation at each stage of the disease [163]. The International Hidradenitis Suppurativa Severity Scoring System (IHS4) is nowadays considered the best scoring system, since it dynamically assesses the severity of the disease, taking into account the number of nodules, abscesses, and draining fistulas/sinus tracts. It stratifies the disease into three severity levels: mild, moderate, and severe, and is useful in both daily clinical practice and clinical trials [8]. Other commonly used scoring systems include the Sartorius score (SS) and the Hidradenitis Suppurativa Physician’s Global Assessment scale (HS-PGA) [164]. The Dermatology Life Quality Index (DLQI) is used to assess the impact of HS on quality of life. Research data consistently show that HS has one of the most significant effects on quality of life [165,166]. The Hidradenitis Suppurativa Quality of Life (HiSQOL) score is a newly developed disease-specific questionnaire that considers features such as drainage and odor [167].

A combination of genetic, immunological, and environmental factors contributes to the complex pathogenesis of HS. Although the first link between genetic factors and HS was suggested in 1985 [168], it was only through recent studies that specific gene mutations were identified. Those mutations affect genes encoding different subunits of γ-secretase, and individuals carrying them usually present with more severe forms of the disease [169]. Inflammation plays a central role in the pathogenesis of HS. The chronic inflammatory response is believed to be triggered by the occlusion of the upper parts of the hair follicle [170]. Hyperkeratosis of terminal follicles, hyperplasia of the follicular epithelium, and perifolliculitis have been observed in histological samples of early HS lesions. The next step is the rupture of the dilated follicle [162,170]. The release of follicle contents into the dermis triggers a rapid and severe immune response involving both innate and adaptive immunity [171]. The skin becomes prone to a Th1/Th17-driven inflammatory response [171], and the proinflammatory cytokines, such as IFN-γ, TNF-α, IL-1, IL-17, and IL-12/23, drive a cycle of inflammation, follicular rupture, and tissue damage [170].

Mechanical stress, smoking, and the cutaneous microbiome are environmental factors that contribute to the pathogenesis of HS. Mechanical stress primarily refers to increased friction in the skin folds [172]. Smoking can significantly worsen symptoms by promoting the activity of proinflammatory cytokines present in HS lesions. Individuals with HS who are current smokers often exhibit a more extensive clinical presentation of the disease compared to non-smokers or former smokers [173]. Although its exact role is still unknown, studies suggest that specific alterations in the skin microbiome may be associated with the disease flares [174].

### The Role of Vitamin D in Hidradenitis Suppurativa

The exact role of vitamin D in HS remains unclear [175]. Vitamin D may play a role in the pathogenesis of HS through its involvement in immune regulation and inflammatory response [176,177]. A study conducted by Ojaimi et al. shows that individuals with vitamin D deficiency had lower levels of toll-like receptor (TLR2) expression [176,178]. When exposed to TLR2 ligands, those participants produced higher levels of proinflammatory cytokines, such as TNF-α and IL-6, than the control group [176,178]. Lee et al. found that vitamin D treatment increased the expression of the antimicrobial peptide LL-37 in cultured sebocytes by activating the VDR. Since antimicrobial peptides contribute to cutaneous innate immunity [179], this suggests that vitamin D may have anti-inflammatory properties [175]. Vitamin D potentially also plays a role in the keratinization pathway. Xie et al. suggested that the interfollicular epidermis and hair follicles require VDR for normal development. Histological analysis of the skin in VDR knockout mice revealed dilated hair follicles and the formation of dermal cysts, with cysts increasing in size and number over time [41,180]. Those changes resemble the early histopathological features of HS [181]. VDR knockout mice also showed a significant reduction in the expression of involucrin, profilaggrin, and loricrin. These proteins are involved in cornified envelope formation and play a role in the process of keratinization [41,180].

Various studies have reported lower vitamin D levels in HS patients compared to healthy controls. The range reported in studies varies widely, reaching up to 100%, depending on the vitamin D levels used as a measure of insufficiency or deficiency [176,182,183,184,185,186,187,188,189,190,191,192]. There is agreement among most studies that there is a correlation between vitamin D deficiency and HS severity, measured by various scoring systems [183,184,186,188,189,191]. A study conducted by Seetan et al. found no significant correlation between vitamin D levels and disease severity [185]. A list of studies that examined vitamin D levels in HS patients and their association with disease severity is presented in Table 5. Few studies have attempted to find a correlation between vitamin D supplementation and its impact on clinical severity of the disease. Fabroccini et al. observed clinical improvement in HS patients who continued their existing therapy with only the addition of oral vitamin D supplementation. Patients with vitamin D insufficiency (21–29 ng/mL) received 25,000 IU of vitamin D per month, while those with vitamin D deficiency (<20 ng/mL) received 50,000 IU per month. The authors concluded that vitamin D supplementation should be considered an integral part of HS management [183]. In a study conducted by Guillet et al., HS patients received between one and six ampoules containing 100,000 IU of vitamin D. Doses were given based on baseline serum vitamin D levels. In 14 out of 22 patients, vitamin D supplementation significantly reduced the number of nodules during the 6-month follow-up [190]. Although promising, it is important to emphasize that these two studies were conducted on a small number of patients. On the other hand, in the study conducted by Guillet et al., supplementation was discontinued in a group of patients who achieved normal vitamin D levels after 3 months [190]. Navarro et al. assessed trabecular bone score (TBS) and bone mineral density (BMD) in HS patients and healthy controls of similar age and sex [188]. Patients with HS showed lower fully adjusted TBS and total hip BMD values. Those values remained significantly lower in patients with HS after adjustment by BMI and tobacco use, therefore suggesting that HS might be an independent risk factor for bone disease [188]. While most studies show that patients with HS have a vitamin D deficiency compared to the healthy population, its role in the pathogenesis of HS is still not fully understood. Although vitamin D is a widely available and inexpensive supplement, research investigating its effect on HS is scarce. Available studies have been conducted on small sample sizes, and a lack of sufficiently long follow-up is common. The studies are not uniformly structured, and to date, no placebo-controlled trials exist. Since most studies have been conducted in tertiary care centers, milder forms of the disease may be underrepresented. This poses a problem, given that vitamin D potentially plays a role in the keratinization pathway, which represents an early stage in the pathogenesis of HS. The question arises whether early supplementation could prevent the cycle of chronic inflammation, altered immune regulation, and consequent structural changes of the skin. Furthermore, no study has proposed which patient subgroups may benefit most from vitamin D supplementation or identified the therapeutic window during which supplementation should be introduced. Further research is needed to fully clarify the role of vitamin D in the pathogenesis and therapy of HS.

## 6. Conclusions

Vitamin D, in addition to its classical role in calcium and phosphate metabolism, is increasingly recognized as a key modulator of skin physiology and immune function. The epidermis is uniquely positioned as both a natural source and a responsive target of vitamin D. Through its active form, 1,25(OH)_2_D and interaction with the VDR, it exerts pleiotropic effects in the skin, including antiproliferative, prodifferentiative and immunomodulatory actions. Vitamin D has multiple roles in the pathogenesis and management of atopic dermatitis, acne vulgaris, psoriasis vulgaris and hidradenitis suppurativa. Its regulatory influence on immune pathways such as Th1, Th17, and Th22, along with its promotion of antimicrobial peptides and involvement in keratinocyte homeostasis, highlights its immunomodulatory and protective potential. Topical vitamin D analogues have demonstrated clinical efficacy, while systemic supplementation offers potential benefits but remains inconclusive. Furthermore, inconsistencies in study outcomes and lack of standardized supplementation protocols require further randomized controlled trials, which should determine optimal dosing strategies and identify patient subgroups that may benefit most from vitamin D supplementation.

## Figures and Tables

**Table 1:** Recent studies on the association between vitamin D serum levels and psoriasis.

Author, Year [Reference Number]	Study Design	*N*	Country	Serum Vitamin D Levels in Psoriasis Compared to Controls	Correlation Between Serum Vitamin D Levels and PASI Scores Without Vitamin D Supplementation	Vitamin D Supplementation: Outcomes
Chandrashekar et al., 2015. [57]	Cross-sectional	43	India	Significantly lower (*p* = 0.004)	Negative correlation (*p* ≤ 0.0001)	N/A
Maleki et al., 2015. [60]	Case-control	50	Iran	Not statistically significant (*p* = 0.06)	No correlation (*p* = 0.03)	N/A
Bergler-Czop et. al., 2016. [61]	Case-control	40	Poland	Significantly lower (*p* = 0.048)	Negative correlation (r = −0.43)	N/A
Pokharel R. et al., 2022. [58]	Case- control	120	Nepal	Significantly lower (*p* = 0.001)	Negative correlation (*p* = 0.01)	N/A
Disphanurat et al., 2019. [59]	RCT	45	Thailand	N/A	Not reported	60,000 IU/week At the three-month follow- up: significant decrease of PASI score (*p* = 0.039) At the six-month follow- up: not significant decrease of PASI score (*p* = 0.16)
Prtina et al., 2021. [69]	Prospective clinical study	40	Bosnia and Herzegovina	N/A	Not reported	5000 IU/day significant improvement (*p* < 0.001)
Ingram et al., 2018. [63]	RCT	101	New Zealand	N/A	Statistically significant (*p* = 0.002)	200,000 IU at baseline, followed by 100,000 IU/month At 12 months: vit. D group and placebo group both showed significantly different PASI scores to baseline; vit D group: (*p* < 0.001), placebo: (*p* = 0.001).
Jarrett et al., 2017. [64]	RCT	65	New Zealand	N/A	Not reported	100,000 IU/month No significant impact on PASI score observed (*p* = 0.71)

Abbreviations: *N*—number of patients with psoriasis, N/A—not applicable, PASI—Psoriasis Area and Severity Index, RCT—randomized controlled trial.

**Table 2:** Summary of studies on vitamin D and atopic dermatitis.

Author, Year [Reference Number]	Study Design	*N*	Country	Serum Vitamin D Levels in AD Compared to Controls	Vitamin D Levels and HS Severity Correlation
El Taieb et al., 2013 [93]	Case-control Study	29 children	Egypt	Lower vitamin D levels in AD patients (*p* < 0.001)	Severity (SCORAD) inversely correlated (*p* < 0.001)
D’Auria et al., 2017 [94]	Case-control Study	52 children	Italy	Lower vitamin D levels in AD patients (*p* < 0.01)	No significant correlation (*p* = 0.31)
Su et al., 2014 [103]	RCT	60 children	Turkey	No significant difference (*p* = 0.065)	Significant correlation (SCORAD) (moderate vs mild *p* = 0.001; severe vs mild *p* = 0.004)
Samochocki et al., 2013 [104]	Cross-sectional Study	95 adults	Poland	No significant difference (*p* > 0.05)	No statistically significant difference
Han et al., 2015 [95]	Cross-sectional Study	72	Korea	Lower vitamin D levels in children with AD (*p* = 0.036), but not in adults with AD (*p* > 0.05)	No significant correlation (*p* > 0.05)
Wang et al., 2014 [96]	Cross-sectional Study	498 children	Hong Kong	Lower vitamin D levels in AD patients	Inverse correlation with both SCORAD (severe vs mild *p* = 0.002; moderate vs mild *p* = 0.011) and NESS (*p* = 0.012)
Munawwarah et al., 2018 [101]	Cross-sectional Study	26 children	Indonesia	N/A	Significant inverse correlation (SCORAD) (*p* = 0.01)
Peroni et al., 2011 [90]	Cross-sectional Study	37 children	Italy	N/A	Significant inverse correlation (SCORAD) (*p* = 0.002)

Abbreviations: *N*—number of AD patients; N/A—not applicable; RCT—randomized controlled trial.

**Table 3:** Vitamin D supplementation and atopic dermatitis—summary of studies.

Author, Year [Reference Number]	Study Design	*N*	Country	Serum Vitamin D Levels in AD Compared to Controls	Vitamin D Supplementation (Dosage)	Vitamin D Supplementation Outcomes
Camargo et al., 2014 [109]	RCT	107 children	Mongolia	Not reported	1000 IU/day for 1 month	SCORAD scores improved (*p* = 0.03)
Mansour et al., 2020 [107]	RCT	86	Egypt	N/A	1600 IU/day for 12 weeks	Significant EASI score reduction compared to placebo group (*p* < 0.001)
Amestejani et al., 2012 [110]	RCT	60	Iran	N/A	1600 IU/day for 60 days	Significant improvement (SCORAD, TIS) compared to placebo group (*p* < 0.05)
Sidbury et al., 2008 [111]	RCT	11 children	USA	Not specified	1000 IU/day for 1 month	Significant improvement (IGA score) in treatment group vs placebo group (*p* = 0.04)
Samochocki et al., 2013 [104]	Cross-sectional Study	95 adults	Poland	No significant difference (*p* > 0.005)	2000 IU/day for 3 months	Significant SCORAD score reduction (*p* < 0.05)

**Table 4:** The association between vitamin D serum levels and acne.

Author, Year [Reference Number]	Study Design	*N*	Country	Significant Findings	Correlation Between Vitamin D Levels and Acne Severity
Lim et al., 2016 [149]	Case-control study	80	Korea	A total of 48.8% of acne patients had vitamin D deficiency compared to 22.5% of controls (*p* = 0.019). Between the groups, there were no differences in the mean vitamin D concentration (*p* = 0.112).	Significant, negative correlation (*p* < 0.001) *
Singh et al., 2021 [148]	Cross-sectional observational study	50	India	Vitamin D deficiency was found in 28% of acne patients and 6.7% of controls (*p* = 0.022). No statistically significant difference in serum vitamin D levels in acne patients and controls (*p* = 0.066).	Significant, negative correlation (*p* < 0.001) *
Kemeriz et al., 2019 [150]	Single-centered, prospective, and controlled study	134	Turkey	Vitamin D deficiency in acne patients was 77.6%, and in control group, 63.8% (*p* = 0.041). There was a significant difference in serum vitamin D levels between the acne group and controls (*p* < 0.001).	Significant, negative correlation (*p* < 0.001) *
Abd-Elmaged et al., 2020 [147]	Case-control, hospital-based study	135	Egypt	Significantly lower levels of 25-OH D3 were noted in acne patients compared to controls. (*p* < 0.05).	Significant, negative correlation (*p* < 0.05) *
Alhetheli et al., 2020 [151]	Cross-sectional study	68	Saudi Arabia	Serum vitamin D levels were significantly higher in the control group compared to the acne group (*p* = 0.003).	Nonsignificant (*p* = 0.067) *
Toossi et al., 2015 [152]	Cross-sectional study	39	Iran	Deficiency of vitamin D was found in 89.7% of acne patients and 90% of controls, with no statistically significant difference (*p* = 0.97). There was no significant difference in the median serum concentrations of vitamin D between the two groups (*p* = 0.14).	Nonsignificant (*p* = 0.45) *
El-Ramly et al., 2016 [153]	Cross-sectional study	60	Egypt	3.3% of acne patients and 11.7% of controls were deficient in vitamin D, with no significant difference between the groups (*p* = 0.141). No statistically significant difference was noted in the vitamin D levels between the acne group and the controls (*p* = 0.226).	Nonsignificant (*p* = 0.95) **

Abbreviations: *N*—number of acne patients, 25-OHD_3_—25-hydroxyvitamin D_3_. * Severity calculated according to the Global Acne Grading System (GAGS). ** Severity calculated according to the Lehmann et al. [154] grading system for severity of acne.

**Table 5:** The association between vitamin D serum levels and hidradenitis suppurativa.

Author, Year [Rference Number]	Study Design	*N*	Country	Significant Findings	Correlation Between Vitamin D Levels and HS Severity
Fabbrocini et al., 2021 [183]	controlled cohort study	40	Italy	Vitamin D deficiency in 77.5% of the patients with resistant HS. Clinical improvement in 27 out of 36 patients with vitamin D supplementation.	Significant, assessed by Sartorius score (*p* < 0.01).
Navarro et al., 2022 [188]	prospective case-control study	81	Spain	Lower vitamin D levels in patients with HS, even after adjusting for age, sex, BMI, fat percentage, diabetes mellitus, estimated GFR, CRP, and the month of the year (*p* = 0.025). Measurements of total hip BMD (*p* = 0.013) and TBS (*p* = 0.007) suggest that HS might be an independent risk factor for bone disease.	Not significant (*p* = 0.07).
Seetan et al., 2022 [185]	comparative cross-sectional study	110	Jordan	All patients (100%) had low vitamin D levels, compared to 65.5% of controls with low levels and 20% with vitamin D insufficiency (*p* < 0.001). Low vitamin D levels, aside from the disease itself, could be explained by inadequate sun exposure.	Not significant (*p* = 0.406).
Moltrasio et al., 2021 [184]	retrospective cross-sectional study	250	Italy	Vitamin D deficiency in 79.84% of the patients. Significant inverse correlation between vitamin D and CRP levels (*p* < 0.0001).	Significant, assessed by IHS4 (*p* < 0.0001).
Koumaki et al., 2024 [186]	cross-sectional study	136	Greece	Lower vitamin D levels in patients compared to healthy controls (*p* = 0.04). Vitamin D insufficiency in 39% and deficiency in 11% of patients (*p* = 0.03).	Significant, assessed by Hurley stage (*p* = 0.02) and IHS4 (*p* = 0.006).
Toker et al., 2024 [191]	retrospective chart review	198	USA	Low vitamin D levels in 38.5% of patients with mild HS and 76.2% of patients with severe HS (*p* < 0.001). Significant differences in the CRP, ESR, and IL-6 levels (*p* < 0.001).	Significant, assessed by HS-PGA (*p* < 0.001).
Sánchez-Díaz et al., 2021 [189]	cross-sectional study	50	Spain	Inverse association between vitamin D levels and number of affected areas in univariate (*p* = 0.002) and multivariate (*p* = 0.047) analysis.	Significant, assessed by IHS4 (*p* = 0.014).
Guillet et al. 2015 [190]	pilot study	22	France	All patients (100%) had a vitamin D deficiency compared to 91% of healthy controls. A total of 36% of patients had a severe vitamin D deficiency (< 10 ng/mL) compared to 14% of healthy controls. Decrease in the number of nodules at 6 months with vitamin D supplementation (*p* = 0.01133).	Significant, assessed by Hurley stage (*p* = 0.03268).
Brandao et al., 2020 [180]	case series	5	Italy	All patients with syndromic HS (PASH and PAPASH) had low vitamin D and elevated CRP and ESR values.	N/A
Kelly et al., 2014 [192]	cross-sectional study	16	Ireland	75% of patients had vitamin D levels < 50 nmol/L. No significant correlation between vitamin D levels and CRP (*p* = 0.66).	N/A

Abbreviations: BMD—bone mineral density, BMI—body mass index, CRP—C-reactive protein, ESR—erythrocyte sedimentation rate, GFR—glomerular filtration rate, HS—hidradenitis suppurativa, IHS4—International Hidradenitis Suppurativa Severity Scoring System, IL—interleukin, N—number of HS patients, N/A—not applicable (HS-specific scores were not assigned to the patients, or the researchers did not investigate the correlation between vitamin D levels and disease severity), PAPASH—pyogenic arthritis + pyoderma gangrenosum + acne + hidradenitis suppurativa, PASH—pyoderma gangrenosum + acne + hidradenitis suppurativa, PGA—Physician’s Global Assessment scale, TBS—trabecular bone score.

## Abbreviations

The following abbreviations are used in this manuscript:

1,25(OH)2D	1,25-dihydroxyvitamin D
25(OH)D	25-hydroxyvitamin D
7-DHC	7-dehydrocholesterol
AD	Atopic dermatitis
AMPs	Antimicrobial peptides
BMD	Bone mineral density
BMI	Body mass index
BPO	Benzoyl peroxide
CaR	Calcium receptor
CRP	C-reactive protein
CYP27A1	25-hydroxylase
CYP27B1	1α-hydroxylase
DLQI	The Dermatology Life Quality Index
EASI	Eczema area and severity index
ESR	Erythrocyte sedimentation rate
GFR	Glomerular filtration rate
HiSQOL	Hidradenitis Suppurativa Quality of Life
Hs	Hidradenitis suppurativa
HS-PGA	Hidradenitis Suppurativa Physician’s Global Assessment scale
IHS4	International Hidradenitis Suppurativa Severity Scoring System
MED	Mediator complex
MMP	Matrix metalloproteinase
*N*	Number of patients
NA	Not applicable
PAPASH	Pyogenic arthritis + pyoderma gangrenosum + acne + hidradenitis suppurativa
PASH	Pyoderma gangrenosum + acne + hidradenitis suppurativa
PASI	Psoriasis Area and Severity Index
PGA	Physician’s Global Assessment scale
PI3K	Phosphatidyl inositol 3 kinase
PIP5K1α	Phosphatidyl inositol 4-phosphate 5-kinase 1α
PLC-γ1	Phospholipase C-γ1
RCT	Randomized controlled trial
SCORAD	Severity Scoring of Atopic Dermatitis
SRC	Steroid Receptor Coactivator
SREBP-1	Sterol regulatory binding protein-1
SS	Sartorius score (SS)
TBS	Trabecular bone score
TEWL	Trans epidermal water loss
TNF-α	Tumor necrosis factor-α
VDR	Vitamin D receptor
